# Plant ER-PM Contact Sites in Endocytosis and Autophagy: Does the Local Composition of Membrane Phospholipid Play a Role?

**DOI:** 10.3389/fpls.2019.00023

**Published:** 2019-01-25

**Authors:** Pengwei Wang, Patrick J. Hussey

**Affiliations:** ^1^Key Laboratory of Horticultural Plant Biology (MOE), College of Horticulture and Forestry Sciences, Huazhong Agricultural University, Wuhan, China; ^2^Department of Biosciences, Durham University, Durham, United Kingdom

**Keywords:** endoplasmic reticulum, ER-PM contact sites, VAP27 proteins, endocytosis, autophagy

## Plant EPCS Resident Proteins

Direct interaction between the ER and PM was identified in plants in many early studies (Staehelin and Chapman, 1987; Staehelin, 1997; Perico and Sparkes, 2018), where two membranes of distinct organelles are in close apposition without fusing. However, proteins localized to these sites have recently been identified in plants and these include VAP27, NET3C, and SYT1 (or SYTA) (Wang et al., 2014, 2016; Pérez-Sancho et al., 2015, 2016; Siao et al., 2016; McFarlane et al., 2017). Of these three proteins, VAP27 is the most well-known (as Scs2 in yeast and as VAP in animals). VAP-like proteins have been shown to be involved in the formation of different MCS as well as EPCS (Salvador-Gallego et al., 2017). The VAP27 family is expanded in plants; for example, the Arabidopsis genome encodes 10 VAP27-related proteins (Wang et al., 2016), while the human and *S. cerevisiae* genomes encode three and two VAP27-related proteins, respectively. Therefore, the function of the plant VAP27 proteins are likely to be more diverse, and they may fulfill some of the functions of those animal EPCS proteins that are missing in plants.

The extended-synaptotagmins (E-SYTs) have also been shown to be essential for the formation of EPCS. The plant SYT family contains five members, all of which are similar in peptide sequence to both synaptotagmins (SYTs) and extended-synaptotagmins (E-SYTs) in metazoans (Manford et al., 2012; Malmersjö and Meyer, 2013). However, plant SYT1 is more functionally equivalent to E-SYTs as knock-out expression affects both ER morphology and PM tethering (Levy et al., 2015; Siao et al., 2016; McFarlane et al., 2017).

NET3C is a member of the NETWORKED family which is unique to plants (Deeks et al., 2012). NET3C localizes to the EPCS and interacts with VAP27 (Wang et al., 2014). Different members of the NETWORKED family bind to the actin cytoskeleton localizing the network to different membranes structures where they act as membrane-cytoskeleton adaptors or linkers. Some other Arabidopsis NET proteins also localize to stationary foci at the PM (e.g., NET2A), these structures may also represent EPCS (Duckney et al., 2017) but further studies are required for this to be confirmed.

In summary, the ER-PM connection is a conserved link observed across phylogeny, with some features that appear to be specific for plants. Because of this conservation, it is likely that many of the known functions of EPCS are likely to be conserved, such as the regulation of phospholipid homeostasis, endocytosis, and autophagosome formation, but the differences may also reflect some plant specific additions/adaptations to their function.

## EPCS in regulating Lipid Transport, Composition, and Homeostasis

Early studies demonstrated that the yeast EPCS resident protein, VAP, binds to lipid binding proteins and recruits these proteins (e.g., Osh2, 3) to the ER-PM interface, where they regulate lipid metabolism (Loewen and Levine, 2005). In addition, many other lipid-synthesizing enzymes are enriched at these contact sites and these include OPI3 (a phosphatidylethanolamine *N*-methyltransferase) which synthesizes phosphatidylcholines (PC). Disrupting the ER-PM connection by knocking-out VAP (Scs2) gene expression reduces PC levels, indicating that an intact EPCS structure is important for the function of OPI3 (Tavassoli et al., 2013). Moreover, lipid synthesis enzymes are not only localized to the EPCS as they may also regulate the formation of this connection, for example, PAH1 (a phosphatidic acid phosphatase enzyme) whose over-expression can restore the ER-PM connection in the *Scs2* mutant (Tavassoli et al., 2013). In Hela cells, on the other hand, EPCS localized lipid binding protein, TMEM24, regulates the transport of phosphatidylinositol between ER and PM (Lees et al., 2017). Similar activities have been reported for many lipid binding proteins in other cell types; such as ORPs/Osh proteins which bind to oxysterol as well as to phosphoinositide (Saheki and De Camilli, 2017; Sohn et al., 2018), and Aster proteins which are recruited to the PM in response to cholesterol accumulation and these proteins transport the excess cholesterol back to the ER (Sandhu et al., 2018).

**Figure 1:**
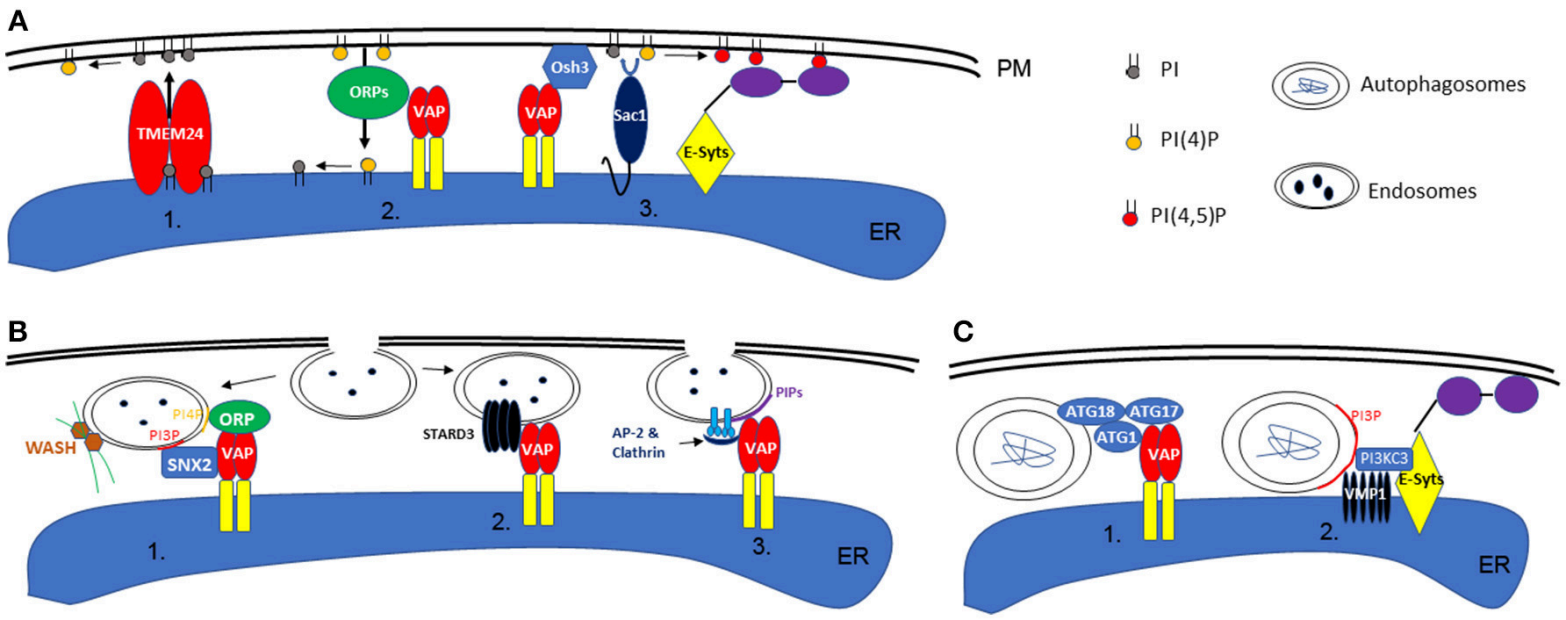
Schematic illustration of phospholipid homeostasis establishment, endocytosis, and autophagy at the ER-PM interface. **(A)** In animal or yeast cells, the local phospholipid content can be achieved by direct transport via TMEM24 (1) or ORP proteins (ORP5 or 8 specifically) (2). Alternatively, this can also be maintained by the activity of Sac1, in concert with VAP or E-Syts at the ER-PM contact sites (3). **(B)** During endocytosis in animals, retromer subunit (SNX2), ORPs, and WASH proteins (activate actin polymerization) are recruited to the endosome membrane by specific phospholipid binding, a machinery that also requires the interaction with VAP (1). On the other hand, ER-endosome interaction is also regulated by VAP-STARD3 interactions (2). Similarly, in plants, the interaction between ER and endosome membrane is also facilitated by VAP proteins and their interaction with Clathrin and AP-2 complex (3). **(C)** In animal cells, the formation of autophagosomes are regulated by EPCS resident proteins VAPs (1) or E-Syts (2), through their interaction with ATG proteins or VMP1.

In addition, another key function of EPCS is to regulate local lipid composition and homeostasis. Among all lipid molecules, phosphatidylinositol serves an essential role in signaling and cytoskeleton re-organization. One of the most well-known regulators of PI-signaling is Sac1, an ER localized PI phosphatase that could covert PI monophosphates (such as PI3P, PI4P) to PI (Nemoto et al., 2000). When activated, Sac1 can be recruited to the PM, where it dephosphorylates PI4P and consequently reduces the level of PI(4,5)P2 in order to maintain steady state (Stefan et al., 2011; Dickson et al., 2016). However, the spatial organization of Sac1 is controlled by EPCS resident proteins, such as E-Syts and the Scs2/Osh3 complex, which interacts directly with the PM when the level of PI(4,5)P_2_ or PI4P is high (Giordano et al., 2013). Furthermore, lipid transfer proteins are also important for regulating lipid homeostasis via EPCS, and the two best examples, from recent studies, are ORP5/8 and TMEM24, which are able to transport PI4P or PI between ER-PM, respectively (Lees et al., 2017; Sohn et al., 2018). This could allow for the replenishment or exchange of PI phosphate at the PM (Figure 1A).

However, the majority of studies in this area are performed in animal and yeast systems. Our knowledge of lipid transport between membrane compartments and the establishment and maintenance of lipid patterning is still lacking in plants (Samuels and McFarlane, 2012). As most of these lipid enzymes are conserved, analogous pathways are likely to exist, but such pathways remain to be investigated.

## Endocytosis likely Occurs at the EPCS in Plants

Once a local phospholipid signature is established, this can be used as a signaling platform to initiate downstream events: the endocytosis pathway for example which can take place at the ER-PM contact sites (Lewis and Lazarowitz, 2010; Wang et al., 2017; Stefano et al., 2018). In Hela cells, VAP proteins interact with STARD3, an endosome localized protein, as well as SNX2, a retromer subunit, mediating the link between ER and endosomes (Alpy et al., 2013; Dong et al., 2016). Such interactions are important in establishing the PI4P composition in endosome membranes and controlling WASH protein (ARP2/3 complex activators of actin nucleation) regulated actin polymerization and endosome budding (Dong et al., 2016). However, the regulatory mechanisms that integrate the ER network, actin cytoskeleton, and endosomes are not very well-understood.

A recent study demonstrates that Arabidopsis VAP27 binds to Clathrin and different forms of PI phosphate including PI(4)P which is enriched in endosomes (Stefano et al., 2018) and PI(3)P which is enriched in autophagosomes (discussed later). When the expression of VAP27-1 and−3 are disrupted, the mutant plants exhibit delayed endocytosis and are defective in endosome internalization (Stefano et al., 2018). It is likely that the ER localized VAP27 can sense PIP-enriched micro-domains on the PM, such as endocytic membranes, and then stay associated. When endosomes are formed at the EPCS, they may stay associated with the ER membrane by interacting with VAP27 and then move along the ER network to different destinations. Therefore, an alteration in plant ER membrane homeostasis will also have a direct effect on the subcellular distribution of endosomes (Stefano et al., 2015). A separate study using another plant EPCS resident protein, SYT1, also demonstrates that overexpressing a dominant negative mutant of SYT1 inhibits the formation of endosomes, further supporting the function of plant EPCS in endocytosis (Lewis and Lazarowitz, 2010).

A similar phenomenon is also seen during non-clathrin-mediated endocytosis (NCE) in Hela cells: the internalization of EGF receptors are regulated by the ER resident protein, reticulon 3 (RTN3). RTN3 co-localizes with E-SYT1 at the EPCS and regulates tubular structure invagination from the PM, in concert with a local calcium ion signal (Caldieri et al., 2017). Interestingly, the interactions between Arabidopsis RNT3, VAP27, and SYT1 have been reported in a proteomics study (Kriechbaumer et al., 2015) and, as such, a similar mechanism of regulation could exist in plants (Figure 1B). No doubt, EPCS-mediated endocytosis is a conserved mechanism in eukaryotic systems, regulated by multiple proteins and likely through redundant pathways.

However, questions remain to be answered. Does endocytosis occur entirely at the EPCS? Is the association between the endocytic site and EPCS dependent on certain conditions and/or on specific stimuli? These questions could be addressed by monitoring endocytosis activity in cells where different levels of ER-PM association are induced; by over-expressing EPCS proteins to create more attachments, or deleting ER-PM tethers to reduce/abolish such interactions. Moreover, future work on the characterization of proteins that regulate ER-endosome interaction will be an intriguing direction to follow in plants as this will potentially give new insight into the function of ER network and post-Golgi trafficking events.

## Could the Formation Of Autophagosomes Take Place at the Plant EPCS?

As discussed previously, a few proteins that localize to the EPCS control local PI phosphate composition and this includes PI3P which is known to be enriched in autophagosome membranes. This feature makes the EPCS a possible location for autophagosome formation. Indeed, a study in Hela cells indicates that the EPCS resident protein, E-Syts, are key regulators of autophagy as aberrant expression of this protein changes the autophagy activity within the cell. E-Syts enhance the ER-PM association when cells are under autophagy stress, and the increased EPCS possibly correspond to enhanced autophagy activity by creating additional sites for autophagosome formation (Molino et al., 2017; Nascimbeni et al., 2017). At these sites, E-Syts interact with VMP1, which stabilizes the PI3KC complex that is required for autophagy-associated PI3P synthesis (Nascimbeni et al., 2017). Moreover, another recent study in COS7 cells further demonstrates the requirement of ER and membrane contact sites for autophagy and where VAP protein is involved. Upon autophagy induction, VAPA/B interacts with multiple ATG proteins, such as WIP2/ATG18, ULK1/ATG1, and FIP200/ATG17, modulating the contact between ER and isolation membrane (IM, the precursor of autophagosomes). Depletion of VAPs impairs the process of autophagosome maturation (Bissa and Deretic, 2018; Zhao et al., 2018; Figure 1C).

In plants, the ER network is also believed to be the key structure for autophagosome formation, but the mechanism of regulation is less well-understood (Zhuang et al., 2017, 2018). For example, the plant ER-PM resident protein, SYT1, may also be involved in autophagy as its KO mutant is more susceptible to a high salt environment, a condition known to trigger autophagy (Schapire et al., 2008). VMP1 (vacuole membrane protein 1), is essential for either E-Syts or VAP regulated autophagy in animal cells. VMP1 interacts with Beclin1/ATG6 during the initiation stages of autophagosome formation (Ropolo et al., 2007). Arabidopsis homologs of VMP1 are known as KMS1 and 2 (Kill Me Slowly), however, our previous study suggested that Arabidopsis KMS proteins do not interact or co-localize with ATG6 under normal growth conditions (Wang et al., 2011), indicating that an alternative regulatory pathway may exist in plants.

## Does the Endocytosis and Autophagy Pathways Converge at the EPCS?

Endosomes and autophagosomes can be formed at the EPCS and a number of studies demonstrate that the fusion between endosomes and autophagosomes occurs prior to their vacuole internalization (Zhuang et al., 2015, 2016). Therefore, it is reasonable to speculate that the conversion/fusion between these two structures can take place at the EPCS. For example, once endosomes are dispatched from the PM, they can interact with VAP27 and stay associated with EPCS (Stefano et al., 2018), where multiple autophagy regulated proteins are also localized (Zhao et al., 2018). Upon the induction of autophagy, endocytic material may be used as the membrane donor for autophagosome biogenesis. However, further experimental evidence is required to test this hypothesis.

## Concluding Remarks

The discovery of MCS and the characterization of molecular regulators required for its formation is a major step forward in cell biology. Each organelle has its unique function, but they also able to interact each other via MCS, forming a complex interaction network. With the help of advanced imaging technique and the combined efforts of scientists from different disciplines, the function of membrane contact sites, especially the EPCS, will become much more evident. With the increasing amount of evidence, plant EPCS are likely to play an important role in endocytosis and autophagy, and this hypothesis will be the subject of future research in this developing field in plants.

## Author Contributions

All authors listed have made a substantial, direct and intellectual contribution to the work, and approved it for publication.

### Conflict of Interest Statement

The authors declare that the research was conducted in the absence of any commercial or financial relationships that could be construed as a potential conflict of interest.
